# 92. Characteristics and Outcomes of Deep Brain Stimulation Device Related Infections: Experience from Quaternary Centers

**DOI:** 10.1093/ofid/ofab466.092

**Published:** 2021-12-04

**Authors:** Hussam Tabaja, Don Bambino Geno Tai, Cristina G Corsini Campioli, Supavit Chesdachai, Daniel DeSimone, Maryam Mahmood

**Affiliations:** Mayo Clinic, ROCHESTER, MN

## Abstract

**Background:**

Increasing use of deep brain stimulation (DBS) over the past 20 years is paralleled by a rise in DBS infections. There is a paucity of data on the diagnosis, management, and outcomes in such infections. We describe our center’s experience with DBS infections.

**Methods:**

Adults ( >18 years) diagnosed with DBS associated infection between January 1, 2000 and May 1, 2020 were retrospectively reviewed. Data on patient demographics, clinical presentation, microbiology, and management was collected.

**Results:**

Seventy cases were identified (**table 1**). The mean age at diagnosis was 58.9 ± 16.5 years. The bulk were free of comorbidities. Parkinson’s disease and essential tremors were the most common indications for DBS placement. The median time from implantation to infection was 4 months [IQR 1,24]. The neurotransmitter and extension wires were the most frequently infected parts. A microbiological diagnosis was made in 89% of cases, 47% of which were polymicrobial. The most commonly identified organisms were *Staphylococcus aureus*, *Cutibacterium acnes*, and coagulase-negative *staphylococci*. For patients with deep infection, 71% had complete device extraction, 20% partial extraction, and 9% device retention; clinical cure at 3 months occurred in 97%, 64% and 100%, respectively (**figure 1**). On the other hand, 93% of patients with superficial infection had device retention; cure at 3 months was seen in 64% (**figure 2**). Suppressive oral antibiotics were rarely used, 45% of patients with partial extraction and 26% with device retention. DBS was reimplanted in 71% of patients after complete extraction and led to reinfection in 30% at 1 year follow up. Median time to reimplantation was 2.7 months. All patients who failed at 3 months in the partial extraction and device retention cohorts subsequently underwent complete device removal leading to clinical cure sustained at 1 year follow up.

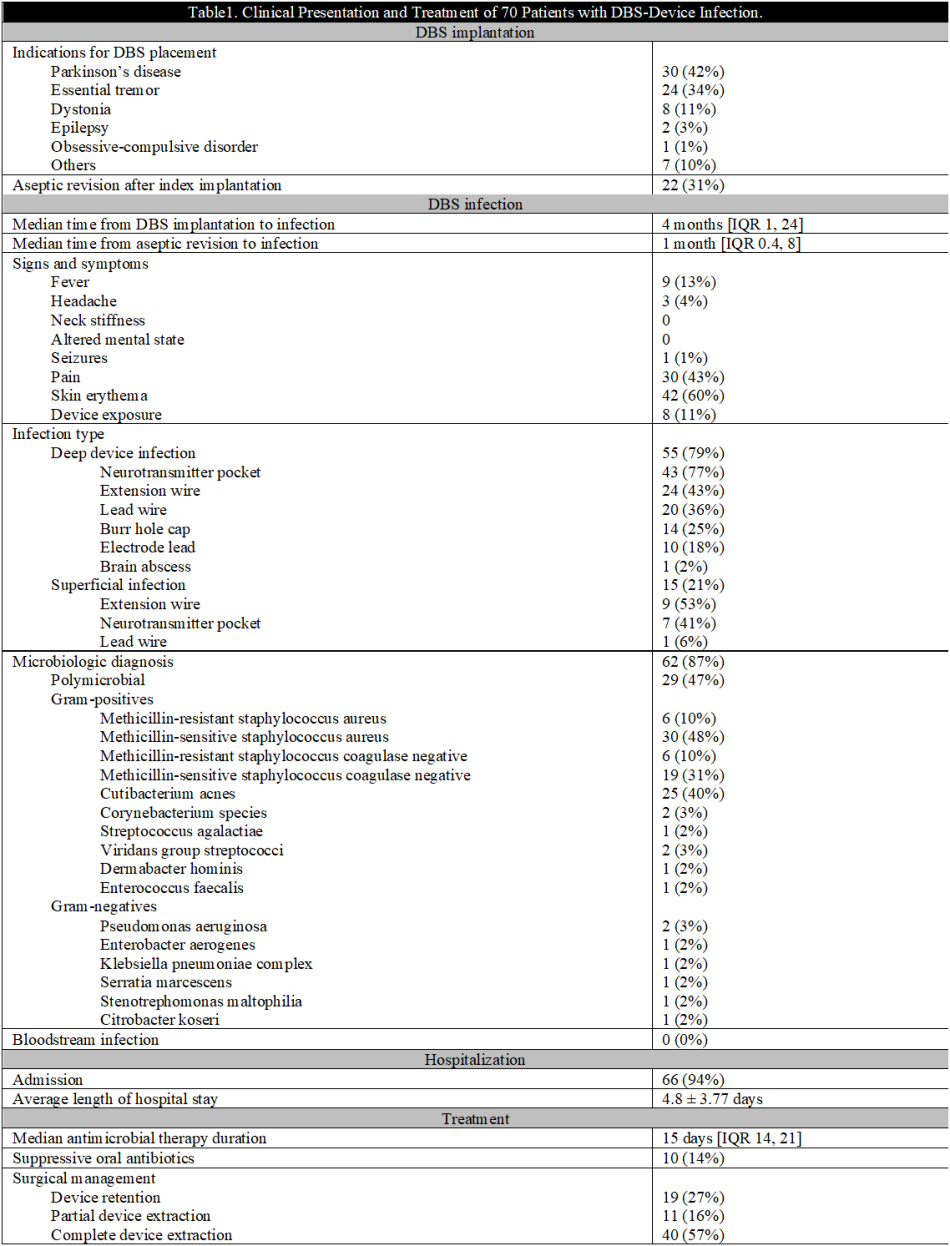

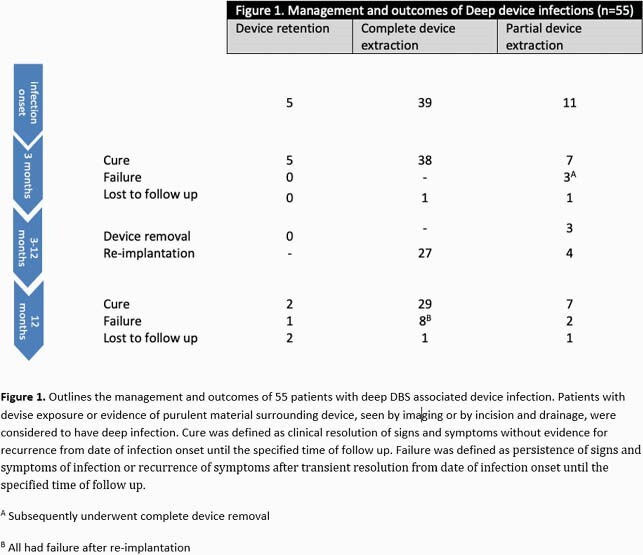

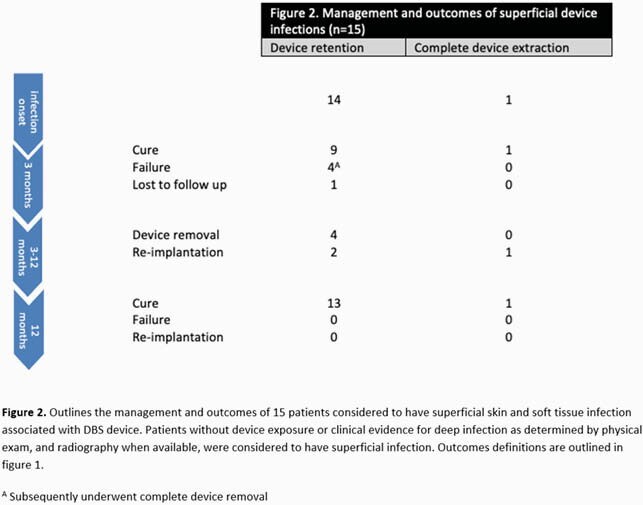

**Conclusion:**

All patients who had complete extraction achieved clinical cure at 3-months follow-up, while high failure rates occurred in those with device retention. Most infections were polymicrobial and predominantly caused by gram-positive pathogens. Thirty percent of patients with re-implantation after complete device extraction developed re-infection within 1 year.

**Disclosures:**

**All Authors**: No reported disclosures

